# On the Treatment of Pericarditis; Especially on the Effects of Bloodletting and Mercury in That Disease

**Published:** 1849-11

**Authors:** John Taylor

**Affiliations:** Fellow of the Royal College of Physicians in London, and Physician to the Huddersfield Infirmary


*On the Treatment of Pericarditis; especially on the Effects of Bloodletting and Mercury in that Disease.* By John Taylor, M. D., Fellow of the Royal College of Physicians in London, and Physician to the Huddersfield Infirmary.—In this communication the author has analysed the forty cases of pericarditis, published in the Lancet, in 1845 and 1846, in respect to the treatment of the disease. The cases are divided into two classes—first, those occurring in connection with acute rheumatism, the subjects of which were previously in good health; and secondly, the cases occurring in connection with renal disease, or in persons previously in a bad state of health.—The patients in the first class, besides being in good health, were younger, and suffered from much fewer complications than those in the second class. Very few of those in the first class died, whereas all died in the second class. The conclusion from these facts is, that the age and previous health of the patients, and the nature of the complicating diseases, have more influence upon the favorable or unfavorable termination of pericarditis than any differences in the treatment. The remedies whose effects are examined are chiefly bloodletting and mercury.

I. *Bloodletting.*—The conclusions arrived at are the following:

1. The duration of pericarditis increases in proportion as the time is longer between the commencement of the disease and the first bleeding.

2. The duration of the cases bled after the first four days is greater by one half than that of those bled within the first four days from the invasion of the disease.

3. The influence of bleeding was more marked in the cases in which it was copiously and repeatedly, as well as early, practised, than in those in which blood was drawn less frequently and more sparingly.

4. Pericarditis is never extinguished at once by bleeding, however early, or however copiously practised.

5. In several cases the pericarditis was suspended for a limited time. The suspension in every instance was immediately consequent upon the local abstraction of blood.

6. It is probable that renal has a longer duration than rheumatic pericarditis.

7. Bloodletting must be less copious, and is more frequently inadmissible, in renal, than in rheumatic pericarditis.

8. Bloodletting probably lessens the mortality, inasmuch as it lessens the duration of pericarditis; but direct proof of the reduction of mortality is not to be obtained from these cases.

9. The abstraction of blood by venesection, cupping, or leeches, almost invariably relieved the pain at once, but not permanently. There is no reason to believe that any one form of bleeding relieved pain more effectually than another.

10. Bloodletting never lessened the frequency of the pulse, except when there were signs of the inflammation having abated.

11. The tendency to syncope in some cases of pericarditis, renders it necessary to be very careful in abstracting blood by venesection.

12. Free venesection for pericarditis does not always prevent the subsequent appearance of serious inflammation in other internal organs.

II. Mercury.

1. The cases in which mercury was given within the first four days had an average duration less by five days than those in which it was given later.

2. The cases in which salivation was produced within the first four days had an average duration less by two days than those in which it occurred later.

3. It is difficult to determine how much of the benefit was due to the mercury, because all the patients who took mercury were likewise bled, and in almost every instance the two remedies were first employed on the same day.

4. The author is inclined to the conclusion, that the benefit was due in greater measure to the bleeding than to the mercury—partly because the duration of the disease was more abbreviated in those who simply began to take mercury than in those in whom salivaton was produced within the first four days. The administration of mercury coincided with the bleeding, but the salivation did not, and the results are just what might be looked for upon the supposition that the benefit was due to the bleeding, and not to the mercury.

5. If the production of salivation had anything like the marked influence in arresting inflammation, and in promoting the removal of its products, which it is currently belived to possess, the duration of the cases of pericarditis after salivation ought to have been much less than it really was. This is proved by a detail of the cases.

(*a.*) Salivation was not followed by any speedy abatement of pericarditis in sixteen cases.

(*b.*) Salivation was followed by pericarditis in five cases.

(*c.*) Salivation was followed by an increase in the extent and intensity of the pericarditis in three cases.

(*d.*) Friction-sound ceased two days before the mouth became sore in two cases.

(*e.*) Salivation was followed by a speedy diminution of the friction-sound in two cases: it did not cease, however, for some days after.

(*f.*) The pericarditis ceased soon after salivation in two cases: in one of them, however, it had been declining for some days before.

(*g.*) Mercury was given, but no salivation was produced in seven cases.

(*h.*) No mercury was given, nor other treatment adopted in eight cases.

(*i.*) Cases are detailed exhibiting the occurrence of various internal inflammations during the time that salivation was proceeding. The cases comprise examples of endocarditis, pleuropneumonia, pneumonia, pleuritis, erysipelas, and rheumatism.

A conclusion rather adverse to the antiphlogistic power of mercury having been drawn from the facts narrated, the author next examines the evidence upon which the contrary and more prevalent opinion is based, and infers that the evidence is not satisfactory. In the course of this examination, some remarks are offered upon the necessity for the application of the "numerical method" in therapeutical inquiries, and, also, upon the difference, and its results, between the practice of French and English physicians, in inflammation of serous membranes.—*Lon. Med. Gaz.*

